# Evaluating the yield of systematic screening for tuberculosis among three priority groups in Ho Chi Minh City, Viet Nam

**DOI:** 10.1186/s40249-020-00766-4

**Published:** 2020-12-09

**Authors:** Luan Nguyen Quang Vo, Andrew James Codlin, Rachel Jeanette Forse, Nga Thuy Nguyen, Thanh Nguyen Vu, Giang Truong Le, Vinh Van Truong, Giang Chau Do, Ha Minh Dang, Lan Huu Nguyen, Hoa Binh Nguyen, Nhung Viet Nguyen, Jens Levy, Knut Lonnroth, S. Bertel Squire, Maxine Caws

**Affiliations:** 1 Friends for International TB Relief, 68B Nguyen Van Troi, 8, Phu Nhuan, Ho Chi Minh City, Viet Nam; 2 Interactive Research and Development, Ho Chi Minh City, Viet Nam; 3 Ho Chi Minh City Public Health Association, Ho Chi Minh City, Viet Nam; 4 Pham Ngoc Thach Hospital, Ho Chi Minh City, Viet Nam; 5 National Lung Hospital, Ha Noi, Viet Nam; 6 KNCV Tuberculosefonds, The Hague, The Netherlands; 7 Department of Global Public Health, Karolinska Institutet, Stockholm, Sweden; 8 Department of Clinical Sciences, Liverpool School of Tropical Medicine, Liverpool, UK; 9 Birat Nepal Medical Trust, Lazimpat, Kathmandu, Nepal

**Keywords:** Case detection, Tuberculosis, Active case finding, Urban, Viet nam

## Abstract

**Background:**

In order to end tuberculosis (TB), it is necessary to expand coverage of TB care services, including systematic screening initiatives. However, more evidence is needed for groups among whom systematic screening is only conditionally recommended by the World Health Organization. This study evaluated concurrent screening in multiple target groups using community health workers (CHW).

**Methods:**

In our two-year intervention study lasting from October 2017 to September 2019, CHWs in six districts of Ho Chi Minh City, Viet Nam verbally screened three urban priority groups: (1) household TB contacts; (2) close TB contacts; and (3) residents of urban priority areas without clear documented exposure to TB including hotspots, boarding homes and urban slums. Eligible persons were referred for further screening with chest radiography and follow-on testing with the Xpert MTB/RIF assay. Symptomatic individuals with normal or without radiography results were tested on smear microscopy. We described the TB care cascade and characteristics for each priority group, and calculated yield and number needed to screen. Subsequently, we fitted a mixed-effect logistic regression to identify the association of these target groups and secondary patient covariates with TB treatment initiation.

**Results:**

We verbally screened 321 020 people including 24 232 household contacts, 3182 social and close contacts and 293 606 residents of urban priority areas. This resulted in 1138 persons treated for TB, of whom 85 were household contacts, 39 were close contacts and 1014 belonged to urban priority area residents. The yield of active TB in these groups was 351, 1226 and 345 per 100 000, respectively, corresponding to numbers needed to screen of 285, 82 and 290. The fitted model showed that close contacts [adjusted odds ratio (a*OR*) = 2.07; 95%* CI*: 1.38–3.11; *P* < 0.001] and urban priority area residents (a*OR* = 2.18; 95% *CI*: 1.69–2.79; *P* < 0.001) had a greater risk of active TB than household contacts.

**Conclusions:**

The study detected a large number of unreached persons with TB, but most of them were not among persons in contact with an index patient. Therefore, while programs should continue to optimize screening in contacts, to close the detection gap in high TB burden settings such as Viet Nam, coverage must be expanded to persons without documented exposure such as residents in hotspots, boarding homes and urban slums.

## Background

Tuberculosis (TB) remains an intractable public health issue. An estimated 10 million persons develop active TB disease and about 1.6 million people die of TB annually [1]. Over the past decade, this disease has garnered greater attention and investment [2–6]. This has led to unprecedented levels of TB patients detected and cured. Nevertheless, declines in TB incidence remain too slow to meet global ambitions to end TB by 2030 [7]. A key reason for this slow rate of decline is the “detection gap”, defined as the 3‒4 million TB patients unreached by national TB control programs (NTP) each year [8]. This gap continues to cause avoidable deaths and fuel transmission of TB [9].

Traditionally, NTPs have relied on passive case finding to detect TB patients. Recognizing that early detection is an essential approach to improve outcomes and abate transmission, the World Health Organization (WHO) recommends systematic screening in key affected populations. However, the number of groups universally recommended for systematic screening remains limited. Strongly recommended groups include household contacts and other close contacts of TB patients, persons living with HIV and persons with silica exposure [10]. Consequently, few persons are actively screened and national programs miss the opportunity to reach more persons suffering from TB [11]. Screening may be expanded to other groups such as urban slum dwellers and migrants, but these recommendations remain conditional [10]. The conditionality derives from the uncertainty that the benefits would outweigh associated costs. This uncertainty is rooted in the lack of documented benefit given the resource intensity of active case finding (ACF) [12]. The paucity of evidence was particularly highlighted among healthcare workers, previously-treated TB patients, and migrants [10].

In Viet Nam, screening for strongly recommended target groups has already been integrated into the country’s relevant national guidelines [13–15]. Screening in household contacts is mainly the responsibility of Viet Nam’s primary care system. Given that TB care and prevention forms only a minor part of their responsibilities, implementation gaps remain and there is evidence that a significant portion of household contacts that develop active TB are missed by routine NTP activities [16]. However, even if household contact investigations for all publicly notified TB patients were fully implemented, the incremental yield of people with TB would amount to about 1% of the national TB burden [16].

Similar to many developing countries, there is a growing trend towards urbanization in Viet Nam. This has resulted in an influx of rural-to-urban migrants into major cities [17]. Ho Chi Minh City (HCMC) is a prime destination for those in search of economic opportunity [18]. A consequence is the continuous growth in temporary and urban poor populations [19]. There is substantial evidence that these populations are at greater risk for TB and poor treatment outcomes [20–22], but it remains unclear whether ACF in these subgroups can yield more case notifications.

Recognizing that routine household contact tracing is both poorly implemented and insufficient to end TB, a community-based ACF project named PROPER CARE was piloted in Go Vap district of HCMC in 2014. This project engaged full-time community health workers (CHWs) for case management and household contact investigation. Project activities further included community-wide door-to-door screening given available CHW capacity. By 2016, the project had increased case notifications by 17.3% over baseline. The majority of cases were found among TB patient contacts, or in presumptive hotspots and areas with high concentrations of slum dwellers and migrants [23]. Based on these results we devised the implementing proven community-based active TB case finding interventions study (IMPACT-TB). One of the study goals was to assess screening yield in these urban priority groups in HCMC, Viet Nam.

## Methods

### Study setting

**Fig. 1:**
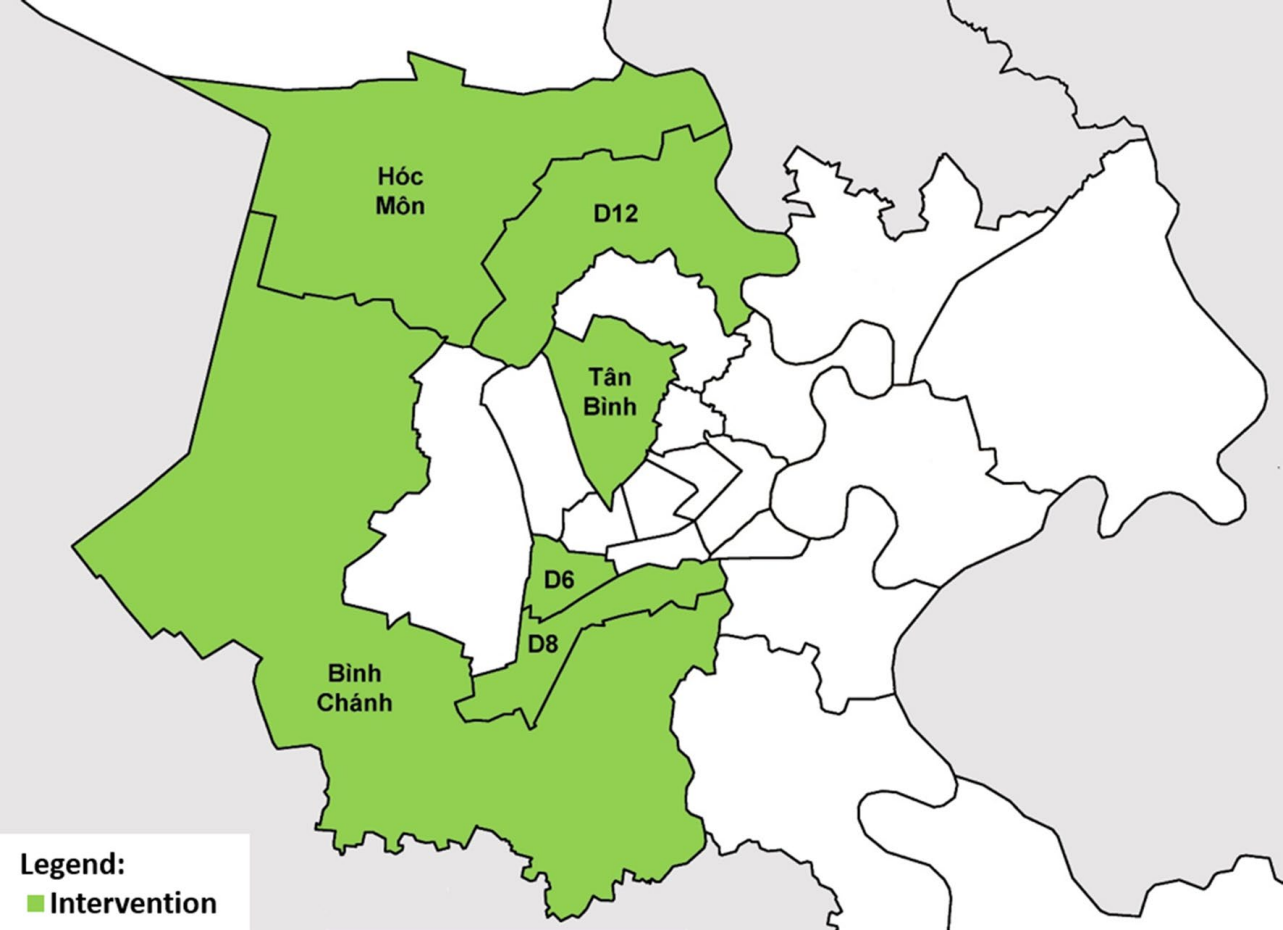
Location of intervention districts in Ho Chi Minh City, Viet Nam

The study was conducted in six of 24 districts of HCMC (Fig. 1) from October 2017 to September 2019, in which we implemented an ACF intervention. The intervention area was selected through consensus with the provincial TB program and public health authorities based on TB burden, absence of confounding interventions and health system readiness to implement the study. It had a population of 2 814 034 and notified 4159 all forms TB patients in 2017 [24]. Each district contained one District TB Unit (DTU), which managed TB diagnosis, treatment and notification according to national guidelines. The Pham Ngoc Thach Provincial TB Hospital (PNTH) provided technical supervision, while the Provincial Health Department and District Health Center served as the administrative authorities.

### Target populations

The study targeted three groups for systematic screening: (1) household contacts of index patients; (2) social, community and close contacts of index patients; and (3) residents of urban priority areas. Index patients were persons treated for any form of TB at one of the DTUs of the intervention area and were notified via the national electronic TB recording and reporting system (VITIMES). Household contacts were defined as persons sharing a kitchen with the index case for one or more nights in the past three months prior to treatment initiation of the index case [25]. Social, community and close contacts, collectively referred to as close contacts hereafter, were defined as having interacted with an index patient at least once per month for any duration of time over the past three months and do not meet the definition of household contact [25]. Residents of urban priority areas, referred to as urban priority groups or urban vulnerable populations hereafter, consisted of persons living in proximity of an index case, in a boarding home or in an urban poor community. Proximity was defined as in a 50 m catchment area of an index case’s residence [26] or the same administrative neighborhood (tổ dân phố) as an index case. Boarding homes were defined as dormitories and single-room rental facilities. The definition of urban poor communities was aligned to United Nations Habitat definitions for slum households [27]. District health authorities aided in the identification and prioritization of boarding home and urban poor communities for screening.

### ACF intervention

**Fig. 2:**
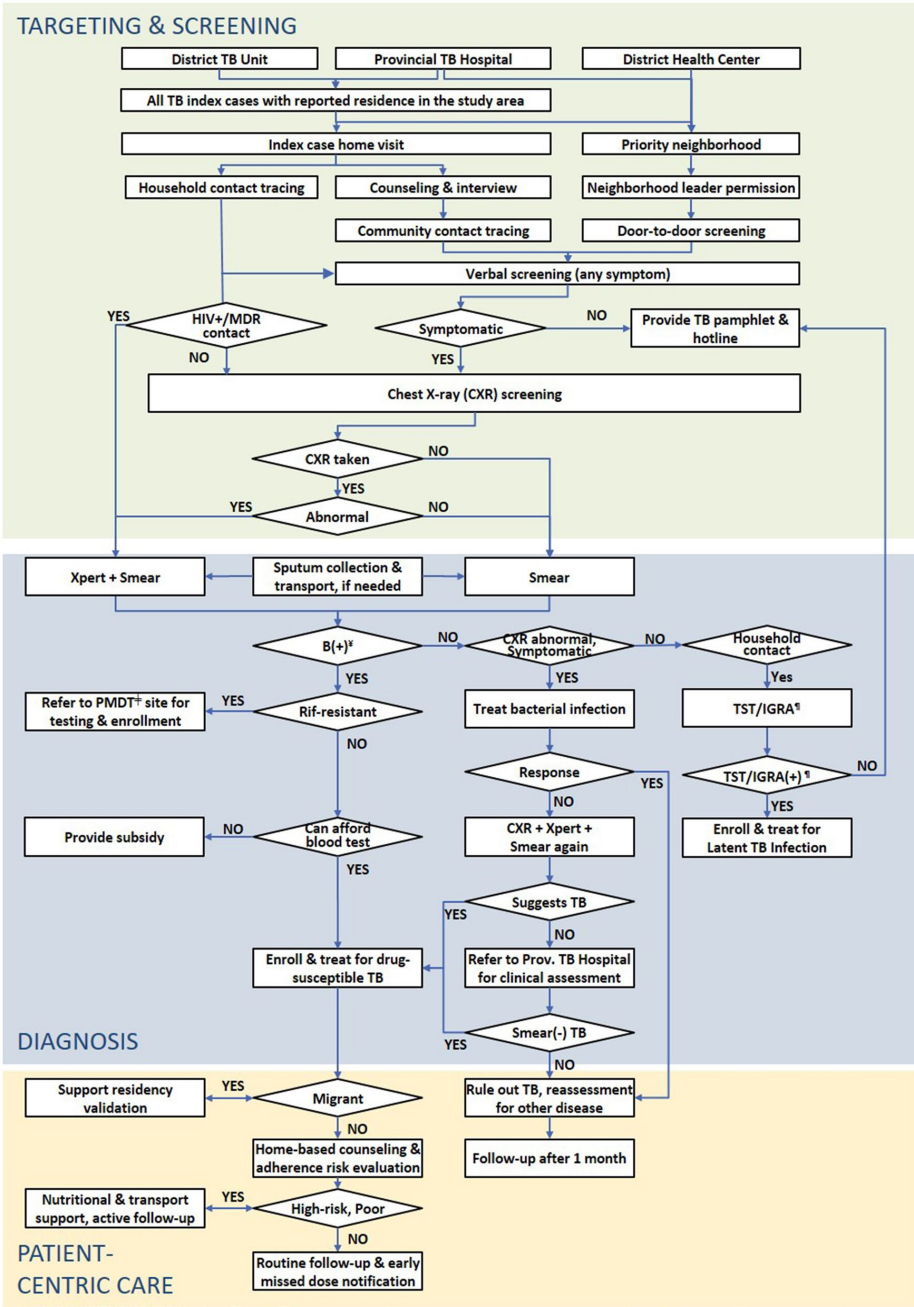
Active tuberculosis case finding algorithm. *TB* tuberculosis, *MDR* multidrug-resistant. ^¥^Bacteriologically-confirmed; ^ǂ^Programmatic management of drug-resistant TB; ^¶^Tuberculin skin test/interferon-gamma release assay

Each intervention district’s local health authorities recruited a cadre of CHW as incentivized volunteers or salaried employees. The differences of these engagement models have been described elsewhere [28]. These CHWs conducted contact investigation and door-to-door screening. Figure 2 shows the ACF intervention applied in the study by target population. The initial screening step consisted of a verbal screen using a bespoke, Android-based mHealth app (TechUp/Clinton Health Access Initiative, Ha Noi, Viet Nam). Verbal screening included history of TB and symptoms such as (productive) cough, hemoptysis, fever, weight loss, night sweats, dyspnea, chest pain, and fatigue. Consent to participate was requested after the verbal screen and before referral for chest X-ray (CXR) screening. Every household contact was referred for a free CXR at their closest DTU or a weekend screening event regardless of their symptomology; others were only referred for CXR if they reported any one of the aforementioned symptoms, had a history of TB or upon request of the individual. Persons with abnormalities suggestive of TB on CXR were tested on the Xpert MTB/RIF assay (Cepheid; Sunnyvale, CA, USA). Symptomatic persons without CXR results or whose CXR result was normal were tested on smear microscopy. Persons with negative sputum test results were evaluated by the DTU and PNTH for clinical diagnosis in accordance to NTP guidelines. Persons diagnosed with active TB were initiated on appropriate treatment.

### Statistical analyses

The primary study outcome was TB treatment initiation. Based on this outcome, we calculated the yield and number needed to screen (NNS) for each target group. We constructed a decision tree to illustrate the TB care cascade [29] disaggregated by the three target populations. The secondary study outcome was a risk factor analysis of covariates significantly associated with the primary study outcome among persons referred for CXR. For this analysis, we described number and proportion of persons eligible for CXR screening and fitted a mixed-effect regression to measure the association between TB treatment initiation as the primary outcome and target group as the primary exposure. We included secondary patient covariates to adjust for confounding. The CHW engagement model and district were the random effects to account for intra-cluster correlation. Due to the unexpectedly high NNS among household contacts and low NNS among close contacts, we developed post-hoc analyses for each group. We conducted an aging analysis for household contacts. This analysis described the time elapsed in monthly intervals between TB treatment initiation of the index patient and the subsequent household contact investigation as well as the treatment initiation of another person in the household with TB. We conducted a two-tailed chi-squared test to test for a significant difference between the two groups in each time interval. For close contacts, we disaggregated the number screened, the number treated for active TB disease, and the NNS into friends, relatives, colleagues, neighbors, classmates and services providers. Similarly, we described the age and gender of treated TB patients as a post-hoc analysis due to the lower risk of TB among older participants. Hypothesis tests were two-sided and point estimates included 95% confidence intervals. Statistical analyses were performed on Stata v13 (StataCorp; College Station, TX, USA).

### Ethical considerations

Ethical approvals were granted by the PNTH Institutional Review Board and the Liverpool School of Tropical Medicine Research Ethics Committee. Study implementation was approved by the HCMC People’s Committee. We obtained written informed consent from participants for inclusion in the risk factor analysis and anonymized patient data prior to analysis. Persons that did not consent still received testing and treatment as per the study protocol and NTP guidelines, but were excluded from the analysis.

## Results

### Study participants

Our sample included 70 439 persons, who consented to be included in the study’s risk factor analysis (Table 1). Women comprised 54.5% of this cohort. The median age was 49 [inter-quartile range (IQR): 33‒62]. Most participants lived in urban districts (63.3%) and had social health insurance (84.9%). Approximately 32.1% had a cough of two weeks or longer, 54.0% reported at least one of the four main TB symptoms of cough, fever, night sweats and weight loss, and 62.5% indicated any symptom suggestive of TB. About 5.8% had a history of TB disease and 7.3% presented abnormalities on CXR. There were notable differences between the target groups. The proportion of female participants was greater among household contacts (58.0%) than among close contacts (50.2%) and urban target groups (52.9%). The median age among household contacts was lower (37; IQR: 22‒53) than among close contacts (44; IQR: 31‒57) and urban target groups (55; IQR: 41‒65). Urban target groups more frequently reported a cough of at least two weeks (42.7%) compared to household contacts (13.2%) and close contacts (26.0%). The pattern was similar for other TB symptoms as well. Urban target groups also more commonly had a history of TB (8.1%) than household contacts (1.9%) and close contacts (2.2%), and showed higher rates of CXR abnormalities (10.2%) than household contacts (2.2%) and close contacts (4.1%).

**Table 1:** Demographic and clinical characteristics of persons screened through active case finding

	Total (*n* = 70 439) *n* (%)	Household contacts (*n* = 23 693) *n* (%)	Close contacts (*n* = 2977) *n* (%)	Urban target groups (*n* = 43 769) *n* (%)
Sex^a^				
Male	31 968/70 211 (45.5)	9877/23 498 (42.0)	1469/2949 (49.8)	20 622/43 764 (47.1)
Female	38 243/70 211 (54.5)	13 621/23 498 (58.0)	1480/2949 (50.2)	23 142/43 764 (52.9)
Age^a^				
< 15 years	4526/68 986 (6.6)	3448/22 899 (15.1)	209/2900 (7.2)	869/43 187 (2.0)
15‒29 years	9543/68 986 (13.8)	5061/22 899 (22.1)	462/2900 (15.9)	4020/43 187 (9.3)
30‒44 years	14 717/68 986 (21.3)	5601/22 899 (24.5)	816/2900 (28.1)	8300/43 187 (19.2)
45‒59 years	19 688/68 986 (28.5)	5509/22 899 (24.1)	831/2900 (28.7)	13 348/43 187 (30.9)
≥ 60 years	20 512/68 986 (29.7)	3280/22 899 (14.3)	582/2900 (20.1)	16 650/43 187 (38.6)
Median age (IQR)	49 (33‒62)	37 (22‒53)	44 (31‒57)	55 (41‒65)
Urbanization				
Peri-urban	25 854 (36.7)	9195 (38.8)	1383 (46.5)	15 276 (34.9)
Urban	44 585 (63.3)	14 498 (61.2)	1594 (53.5)	28 493 (65.1)
Social health insurance^a^				
No	11 046/70 342 (15.7)	3806/23 654 (16.1)	641/2969 (21.6)	6599/43 719 (15.1)
Yes	59 296/70 342 (84.3)	19 848/23 654 (83.9)	2328/2969 (78.4)	37 120/43 719 (84.9)
Cough 2 weeks				
No	47 842 (67.9)	20 559 (86.8)	2203 (74.0)	25 080 (57.3)
Yes	22 597 (32.1)	3134 (13.2)	774 (26.0)	18 689 (42.7)
Four main TB symptoms^c^				
No	32 406 (46.0)	16 682 (70.4)	1614 (54.2)	14 110 (32.2)
Yes	38 033 (54.0)	7011 (29.6)	1363 (45.8)	29 659 (67.8)
Any TB symptoms^b^				
No	26 406 (37.5)	15 748 (66.5)	1508 (50.7)	9150 (20.9)
Yes	44 033 (62.5)	7945 (33.5)	1469 (49.3)	34 619 (79.1)
Previous history of TB				
No/unknown	66 362 (94.2)	23 238 (98.1)	2911 (97.8)	40 213 (91.9)
Yes	4077 (5.8)	455 (1.9)	66 (2.2)	3556 (8.1)
Chest X-ray result				
No chest X-ray	26 529 (37.7)	14 274 (60.3)	1944 (65.3)	10 311 (23.6)
Normal	38 804 (55.1)	8905 (37.6)	910 (30.6)	28 989 (66.2)
Abnormal	5106 (7.3)	514 (2.2)	123 (4.1)	4469 (10.2)

^a^N sizes listed due to missing values

^b^Includes (productive) cough, hemoptysis, chest pain or dyspnea, fever, night sweats, and fatigue of any duration

^c^Includes cough, fever, night sweats and weight loss of any duration

### Active case finding outputs

**Fig. 3:**
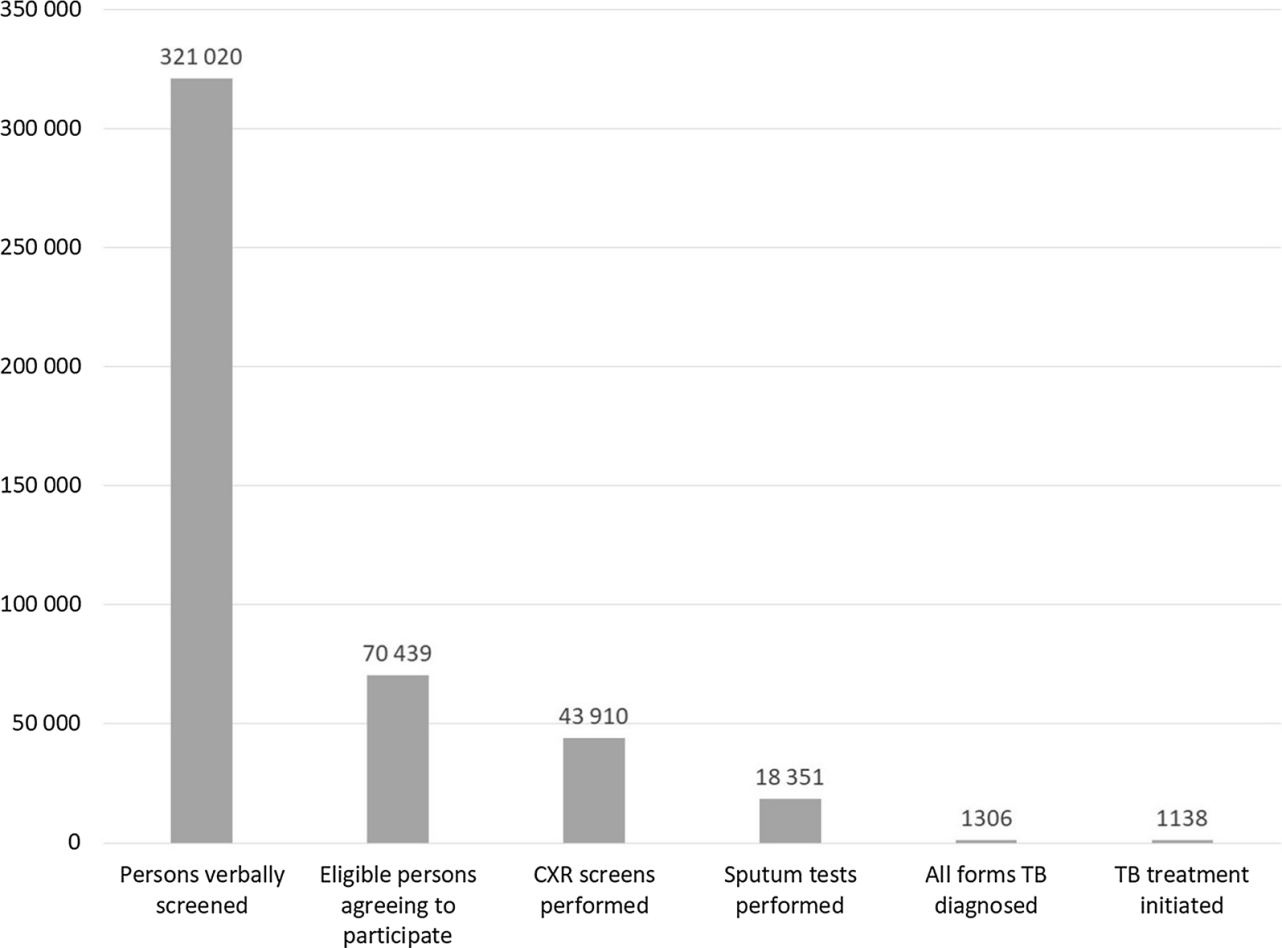
Aggregate tuberculosis care cascade. *CXR* chest X-ray, *TB* tuberculosis

The aggregate TB care cascade is in Fig. 3. Overall, 321 020 persons were verbally screened and 21.9% (70 439/321 020) were deemed eligible for CXR referral and were willing to participate, i.e., present for CXR screening. Among those, 62.3% (43 910/70 439) received a CXR screen and 26.1% (18 351/70 439) a sputum test. All Forms TB was diagnosed in 1306 participants, of whom 87.1% (1138/1306) were linked to care. This corresponded to a yield of 354 per 100 000 in the population screened for a NNS of 282.

**Fig. 4:**
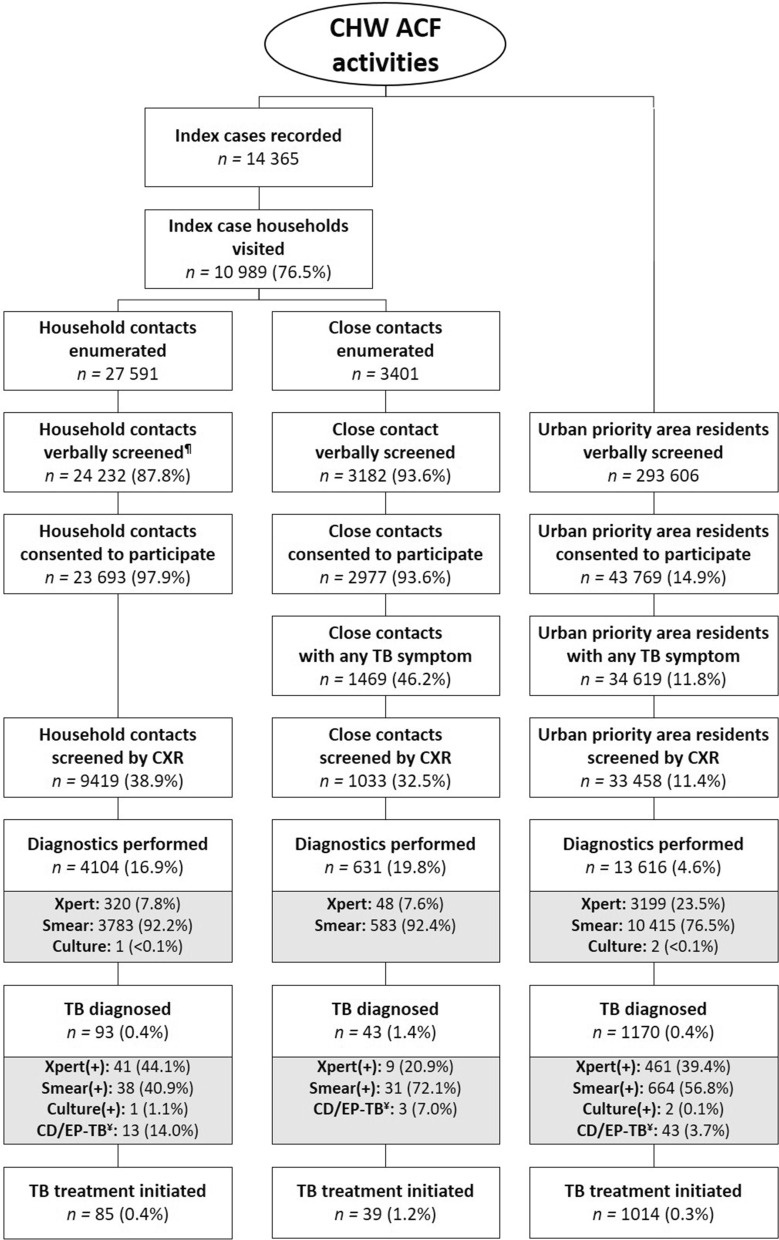
Tuberculosis care cascade by urban priority group. *ACF* active case finding, *CD* clinical diagnosis, *CHW* community health worker, *CXR* chest X-ray, *EP* extra-pulmonary, *TB* tuberculosis. ^¶^Verbal screening for household contacts conducted for study-related data collection only; ^¥^clinical diagnosed/extrapulmonary tuberculosis

The care cascade disaggregated by target population (Fig. 4) shows that over two years the NTP initiated 14 365 all forms TB cases on treatment across the six intervention districts. CHWs visited 10 989 (76.5%) households and enumerated 27 591 household contacts for an average household size of 3.53 persons. CHWs verbally screened 24 232 (87.8%) of the enumerated contacts and 23 693 (97.9%) consented to participate. Active TB disease was diagnosed in 0.4% (93/24 232) of whom 91.4% (85/93) were linked to care. This yield corresponded to a NNS of 285. During household visits, CHWs further enumerated 3401 close contacts for a rate of 0.3 close contacts per index patient. Of these, 3182 (93.6%) were reached for verbal screening and 2977 (93.6%) were eligible and consented to participate. Active TB disease was diagnosed in 1.4% (43/3182) of screened close contacts and 39 (90.7%) were linked to care for a NNS of 82. CHWs further reported that 293 606 residents of urban priority areas were verbally screened and 43 769 (14.9%) were eligible and consented to be included in the analysis. Active TB disease was diagnosed in 1170 (0.4%) persons of whom 86.7% (1014/1170) were started on treatment for a NNS of 290.

### Risk factor analysis

The adjusted odds ratios are shown in Table 2. Close contacts [adjusted odds ratio (a*OR*) = 2.07, 95% *CI*: 1.38–3.11; *P* < 0.001] and residents of urban priority areas (a*OR* = 2.18, 95% *CI*: 1.69–2.79; *P* < 0.001) were more likely to be treated for active TB compared to household contacts. Coughing for two weeks or longer (a*OR* = 2.55, 95% *CI*: 2.12–3.06; *P* < 0.001) and experiencing least one of the four main TB symptoms (a*OR* = 1.52, 95% *CI*: 1.02–2.26; *P* = 0.039) were significantly associated with TB disease. Participants presenting abnormalities on CXR (a*OR* = 76.03, 95% *CI*: 58.72–98.44; *P* < 0.001) and persons without CXR results (a*OR* = 6.18, 95% *CI*: 4.69–8.13; *P* < 0.001) were strongly associated with TB disease in comparison to persons with a normal CXR. Conversely, female participants were significantly less likely to have TB than male participants (a*OR* = 0.55, 95% *CI*: 0.48–0.64; *P* < 0.001). Using the age group of 30‒44 years as referent, persons under 15 years (a*OR* = 0.10, 95% *CI*: 0.03–0.32; *P* < 0.001), between 45‒59 years (a*OR* = 0.77, 95% *CI*: 0.65–0.92; *P* = 0.003) and aged 65 and older (a*OR* = 0.41, 95% *CI*: 0.34–0.49; *P* < 0.001) exhibited a significantly lower association with TB treatment initiation.

**Table 2:** Adjusted associations of urban priority group and secondary covariates with tuberculosis treatment notification from active case finding (*n* = 68 709)

	a*OR*	95% *CI*	*P* value^Þ^
Target group			
Household contacts^¥^	1.00		
Close contacts	2.07	(1.38–3.11)	< 0.001
Urban priority area residents	2.18	(1.69–2.79)	< 0.001
Sex			
Male^¥^	1.00		
Female	0.55	(0.48–0.64)	< 0.001
Age			
< 15 years	0.10	(0.03–0.32)	< 0.001
15‒29 years	0.94	(0.74–1.18)	0.577
30‒44 years^¥^	1.00		
45‒59 years	0.77	(0.65–0.92)	0.003
≥ 60 years	0.41	(0.34–0.49)	< 0.001
Urbanization			
Peri-urban^¥^	1.00		
Urban	1.13	(0.93–1.36)	0.211
Health insurance			
No^¥^	1.00		
Yes	0.87	(0.74–1.03)	0.103
Cough 2 weeks			
No^¥^	1.00		
Yes	2.55	(2.12–3.06)	< 0.001
Four main TB symptoms^§^			
No^¥^	1.00		
Yes	1.52	(1.02–2.26)	0.039
Any TB symptoms^┼^			
No^¥^	1.00		
Yes	1.46	(0.95–2.25)	0.087
Chest X-ray result			
Normal^¥^	1.00		
Abnormal	76.03	(58.72–98.44)	< 0.001
No chest X-ray	6.18	(4.69–8.13)	< 0.001
Previous history of TB			
No^¥^	1.00		
Yes	0.90	(0.76–1.08)	0.259

*aOR* adjusted odds ratio, *CI* confidence interval

ÞWald test

^¥^Referent

^┼^Includes (productive) cough, hemoptysis, chest pain or dyspnea, fever, night sweats, and fatigue of any duration

^§^Includes cough, fever, night sweats and weight loss of any duration

### Secondary and post-hoc analyses

**Fig. 5:**
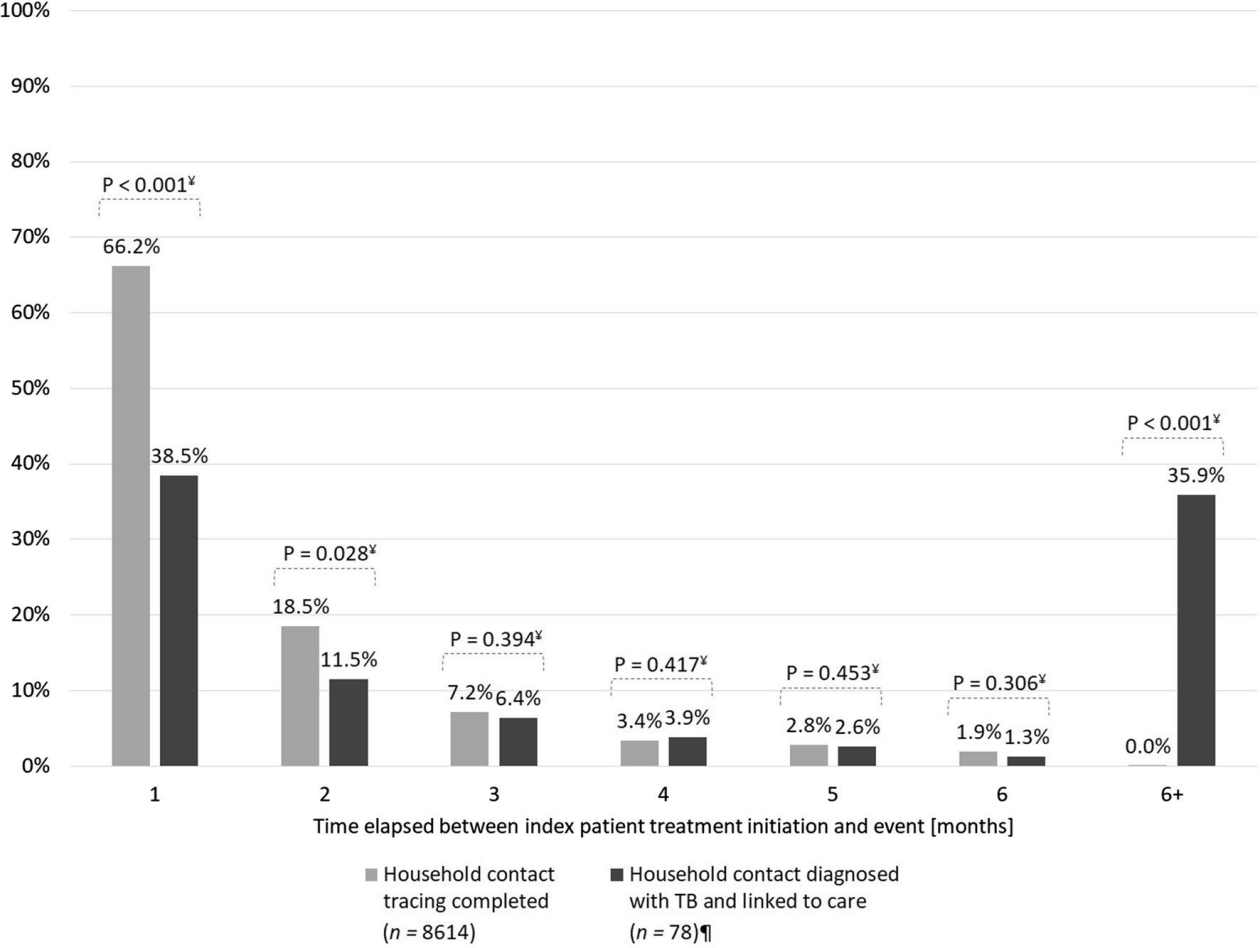
Time interval from index patient treatment initiation to household contacts investigation and treatment initiation of a household contact with tuberculosis. ^¥^Two-tailed χ^2^ test; ^¶^exact treatment enrollment date for 7 index cases not available

The aging analysis assessing the time interval from index patient treatment initiation to contact investigation (Fig. 5) showed that 66.2% of TB patient households were visited within the first month after treatment enrollment. Another 18.5% of patients were visited during the second month of treatment for a total of 84.7% during the intensive phase. We detected only 40.0% of TB cases among household contacts within the first two months. Meanwhile, in 35.9% of household contacts detected with TB, the index case had been treated more than six months prior to the contact tracing event.

Of the 2977 close contacts screened, the index patient had specified the relationship for 2899 (97.4%) (Table 3). Of the subsequently screened close contacts, 1619 (56.6%) were neighbors, 831 (23.1%) were relatives, 236 (8.3%) were friends. Among the 39 close contacts treated for TB, neighbors comprised the largest subgroup with 35 TB patients (89.7%) for an NNS of 46. There were three TB treatment initiations (7.7%) among relatives for an NNS of 277. One treated TB case (2.8%) was a friend of an index case for an NNS of 236.

**Table 3:** Types of close contact screened and treated for active tuberculosis

	Screened^¥^ (*n* = 2899) *n* (%)	Treated for TB^┼^ (*n* = 39) *n* (%)	Number needed to screen *n*	Screening yield (per 100 000)
Close contact type				
Neighbor	1619 (56.6)	35 (89.7)	46	2162
Relative	831 (29.1)	3 (7.7)	277	361
Friends	236 (8.3)	1 (2.6)	236	424
Coworker	134 (4.7)	0 (0.0)	–	–
Service provider	37 (1.3)	0 (0.0)	–	–
Classmates	3 (0.1)	0 (0.0)	–	–

– not applicable

^¥^78 (2.6%) close contacts missing information on type of relationship

^┼^All forms tuberculosis (TB), i.e., bacteriologically-confirmed and clinically diagnosed TB patients

## Discussion

Our study showed that systematic screening in three urban priority groups using community health workers can yield a substantial number of persons with active TB. We further illustrated the necessity to expand beyond contact investigation to achieve higher coverage and find more people with TB in the community. Nine of ten people diagnosed with TB in this study came from screening in persons without clear prior exposure to TB. The obvious downside was the high number of people that needed to be screened in order to detect and treat a person with active TB. Even though the NNS in this group (290) was lower than indiscriminate community screening in comparable settings (weighted mean NNS = 603) [30], this NNS was still on order of magnitude higher than that of strongly recommended groups. Therefore, it is necessary to optimize screening in persons without clear prior exposure to TB or silica, or clinical risk factors such as HIV.

Our study achieved a high yield in close contacts of index cases supporting the recommendation to target this population for ACF. The NNS obtained from our study (82) was similar to previously reported results (weighted mean NNS = 85) [30]. Our data further showed that index cases most commonly referred neighbors to targeted screening. This group contained nine of ten active TB cases initiated on treatment. This is concordant with prior analysis showing the relevance of neighborhood contacts as potentially vulnerable populations for targeted screening [31, 32]. A prior analysis has shown that the ability to identify hotspots a priori can significantly improve door-to-door screening yield in catchment areas around index cases [26]. As such, door-to-door screening in neighborhoods that house both an index case and a referred neighborhood contact may be a way to sustain high yields while expanding coverage.

However, as only one close contact was enumerated for every three index patients engaged, the coverage achieved among close contacts was limited. A key barrier was the reluctance of index patients to identify others in their social network for targeted screening. Stigma and discrimination were likely reasons for this reluctance [33, 34]. Additional barriers could be the religious and sociocultural context of Buddhism and Confucianism in Vietnamese culture. This cultural context has been associated with passiveness and acceptance of suffering as a normal part of life [35, 36]. Past studies have observed Vietnamese people to hide the extent of their suffering and delay health-seeking [37]. Future close contact tracing should include advocacy tailored to the socio-cultural context to improve participation and referrals [38, 39].

Even though it was more than twice the estimated national incidence rate (182/100 000) [40], our study produced the lowest yield among household contacts. One reason was the lower rate of TB-related symptoms. This was in part due to the study’s diagnostic algorithm, which indicated CXR screening for all enumerated household contacts irrespective of their clinical presentation. As a result, the cohort of household contacts included fewer sick persons and referrals for a CXR were only successful for 39.8% of household contacts compared to 70.3% for symptomatic close contacts and 96.6% of urban target groups. Anecdotal feedback from household contacts substantiated this discrepancy, as these asymptomatic contacts were less motivated to present for CXR screening. Conversely, the high CXR conversion rate in the other two populations was likely a result of the higher value perception of CXR given the positive verbal pre-screening. While this result shows the value of verbal pre-screening, it may contribute to under-diagnosis of cases. This is particularly the case given the high rate of TB patients without symptomatic presentation as observed on both prevalence surveys [41, 42].

A second reason related to the demographics of screened household contacts. Household contacts were both younger and more often female. Viet Nam’s TB prevalence surveys showed that prevalence of TB was lower in younger populations, while older populations are another conditionally recommended group for screening [10]. TB prevalence has historically been higher among men [43], which studies have linked to the dichotomy in occupational and behavioral differences across genders in Viet Nam [44], although the complex underlying causes remain insufficiently elucidated.

Another reason for the high NNS in household contacts may relate to the study’s contact tracing procedures. CHWs were asked to visit households within one month of treatment initiation to combine contact tracing and adherence counseling. As the aging analysis showed high implementation fidelity, the low yield among household contacts may have been a negative by-product. This effect has been previously observed in Viet Nam. Household contact tracing on treatment initiation yielded only 26.7% of eventually detected TB cases in the household and at six months the yield was 57.2% [45]. Our observed yields by time elapsed until contact investigation are concordant with those results. We did not offer TB preventive treatment (TPT) to eligible household contacts. This was a limitation of our approach, due to funding constraints, and future efforts should consider the yield of active TB and simultaneous TPT provision as well as future active TB averted due to TPT provision in their NNS calculations.

Our risk analysis supported the finding that systematic screening should be intensified in close contacts and urban priority area residents. Both groups were more closely associated with the case detected yield in our study. The analysis also showed that cough of two weeks or more remains a strong predictor of TB disease, while the presence of the four main TB-related symptoms was moderately associated with TB disease. Conversely, screening for the presence of additional symptoms such as chest pain and dyspnea was not associated with yield. Similarly, in our study older persons were less associated with case detection yield. This was possibly a function of the door-to-door screening among urban priority populations, which generally occurred during the day. During these screening encounters, CHWs reached a greater proportion of older persons, many of whom did not have TB. Instead, the community-based ACF activities detected a large proportion of working age men with TB (Additional file 1, Table S1), who comprise a key unreached vulnerable population in Viet Nam [42].

CXR abnormality was another strong indicator of risk of TB. This was likely a result of employing the double-X algorithm, which enabled access to the more sensitive Xpert test as the initial diagnostic tool in this cohort compared to smear microscopy in other persons tested. This is concordant with other settings and supports the NTP’s country-wide programmatic expansion of the algorithm [46–48]. Even though the relative risk was lower, the cohort without CXR results was also strongly associated with TB treatment initiation compared to persons with a normal CXR. This was likely due to the inclusion of persons who would have presented TB-related abnormalities, had they taken a CXR. As we conducted our study in a programmatic setting, the large number of smear microscopy tests further illustrates the need to broaden access not only to Xpert, but also to CXR in order to justify resource-intensive ACF activities.

The expansion of TB service coverage is critical for closing the detection gap and reaching the End TB goals [49]. Contact investigation undoubtedly should remain the primary adjunct activity to routine program activities. However, the cases detected in this study would have been a fraction without the inclusion of vulnerable groups absent a clear transmission linkage to an index case. Hence, it will be important to conduct studies to optimize and further illustrate the benefits of screening in conditionally recommended priority groups. It will also be necessary to conduct cost-effectiveness assessments that can delineate the trade-offs between the focus on short-term savings and the avoidance of economic loss and human suffering inherent to the long-term elimination of TB.

As the selection of the intervention districts was purposive and limited to urban Ho Chi Minh City, and our study employed convenience sampling of persons screened, our results likely contain bias. Appropriately powered cluster randomized trials would provide a more robust evaluation of these issues. As our study was implemented in a programmatic setting, we were exposed to supply chain interruptions, capacity limitations and restrictive operating hours. These exposures at times impaired adherence to the study’s diagnostic algorithm and patient care provision. Furthermore, occasional delays in data entry into the routine surveillance system hindered timely follow-up with index cases. However, these issues reflect the reality of field scale-up of an intervention.

## Conclusions

The study detected a large number of unreached persons with TB, but most of them were not among persons in contact with an index patient, but urban vulnerable populations such as residents of hotspots, boarding homes and urban slums. With a decade remaining until the first major End TB milestone, it is critical to not just optimize screening in strongly recommended target groups such as household and close contacts, but also to expand systematic screening for TB to groups with higher NNS in high TB burden countries such as Viet Nam. Urban priority populations can substantially increase yield and urban strategies to end TB. This group should be further considered for inclusion in systematic screening efforts.

## Supplementary information

**Additional file 1: Table S1.** TB treatment notifications by age and sex

## Abbreviations

ACF: Active case finding
AFB: Acid-fast Bacilli
aOR: Adjusted odds ratio
CD: Clinical diagnosis
CHW: Community health worker
CI: Confidence interval
CXR: Chest X-ray
DTU: District TB unit
EP: Extra-pulmonary
HCMC: Ho Chi Minh City
HIV: Human immunodeficiency virus
IQR: Inter-quartile range
NNS: Number needed to screen
NTP: National TB Control Program
PNTH: Pham Ngoc Thach Provincial TB Hospital
TB: Tuberculosis
WHO: World Health Organization

## Availability of data and material

The data that support the findings of this study are available from the Viet Nam National TB Control Program and Pham Ngoc Thach Provincial TB Hospital, but restrictions apply to the availability of these data. Data are can be made available from the authors upon reasonable request and with permission of the Viet Nam National TB Control Program and Pham Ngoc Thach Provincial TB Hospital.
