# Supplementary material for: Evaluating the yield of systematic screening for tuberculosis among three priority groups in Ho Chi Minh City, Viet Nam
Source: Infect Dis Poverty. 2020 Dec 9;9:166. doi: 10.1186/s40249-020-00766-4 (PMC7724701; doi:10.1186/s40249-020-00766-4)
Supplement: Supplementary file 1 — Additional file 1: Table S1. TB treatment notifications by age and sex [file 40249_2020_766_MOESM1_ESM.docx]

# ADDITIONAL information

## Supplementary results

## Table S1: TB treatment notifications by age and sex

|  | **Total**  **(N = 1,138)** | **Male**  **(N = 855)** | **Female**  **(N = 283)** |
| --- | --- | --- | --- |
| **Age** **(N, %)** |  |  |  |
| <15 years | 3 (100.0) | 3 (100.0) | 0 (0.0) |
| 15-29 years | 124 (100.0) | 64 (51.6) | 60 (48.4) |
| 30-44 years | 293 (100.0) | 218 (74.4) | 75 (25.6) |
| 45-59 years | 411 (100.0) | 339 (82.5) | 72 (17.5) |
| >60 years | 307 (100.0) | 231 (75.2) | 76 (24.8) |
